# In Memoriam: Leonard Mindich 1936–2024

**DOI:** 10.3390/v17111451

**Published:** 2025-10-31

**Authors:** Paul Gottlieb

**Affiliations:** School of Medicine, The City University of New York (CUNY), New York, NY 10530, USA; pgottl@med.cuny.edu


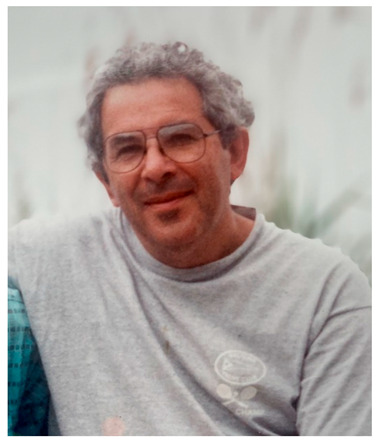


Leonard Mindich passed away over one year ago at his home in New York City at age 88, after a lengthy battle with Parkinson’s Disease that included cardiovascular complications. His death was reported by his family within a short New York Times Obituary that provided biographical information describing Lenny’s background and his wide-ranging interests. At the time, none of the professional journals made a note of his death or provided any type of statement of the loss of a pioneer of molecular virology. The entire early history of cystovirus studies by Lenny and others is currently available in this Special Issue of Viruses. Indeed, this Special Issue likely would not have existed had it not been for Lenny’s research, which encouraged other scientists to study this unique viral family. Lenny’s career was exceptional, in that it spanned the eras of molecular biology, from the advent in the immediate post-war years until our modern age of structural biology. His studies included all the aspects of these times, with the remarkable ability to readily adapt and employ each new technology into his research; he always provided rich and insightful observations [1].

Lenny came from a humble background; he grew up in the Tremont area of the Bronx New York City and attended the academically selective Bronx High School of Science. He once told his lab team that, according to family lore, his father snuck from Canada to New York State as an illegal immigrant by rowboat and then made his modest success in the USA in the laundry business. Lenny attended the Agricultural School of Cornell University, as this academic avenue offered low tuition. He often spent the summer season as a volunteer at the Cold Spring Harbor Laboratories, and there, he closely interacted with the luminaries of the early bacteriophage group, including Max Delbrück, Salvador Luria, and James Watson. There, Lenny met and was taken under the wing of the legendary physicist-turned-biologist Leo Szilard, who he greatly admired but described as an eccentric. He then did his graduate research at Rockefeller University, under the direction of Rollin Hotchkiss. The loose and less-structured curriculum of Rockefeller suited Lenny’s independent nature and he received his PhD degree in 1962. Lenny performed a post-doctoral year at the Pasteur Institute in Paris and joined the Public Health Research Institute of New York (PHRI) in 1962, where he remained for the rest of his career.

PHRI was then located on Manhattan’s Lower East Side, housed on three floors of New York City’s Public Health Laboratory Building on First Avenue. There, Lenny ran a small but extremely productive laboratory, where graduate students, post-doctoral fellows, and even the technicians flourished. Jeffrey Strassman, the laboratory’s senior member, kept us all on an even keel and made sure all ran smoothly. Lenny frequently hosted visiting fellows from the laboratory of Dennis Bamford in Helsinki, Finland in collaborative efforts that produced friends as well as data (sadly, Dennis’s passing was just announced as I am writing this memorial). Lenny’s office door was always open to discussions, where ideas, concepts, and experimental plans were hatched. The day usually opened with Lenny asking his team what was up, or new, using the Yiddish word “Nu”? And there was often much new information to describe.

Lenny had an enduring optimism and always brought out the best in all the staff, including myself. I arrived at the lab and certainly was no “wunderkind”, but I still sequenced two of the three φ6 double-stranded RNA (dsRNA) segments; at the time, this was performed manually by reverse-transcribing RNA, cloning the cDNA segments into bacteriophage M13, and using dideoxy nucleotide chain termination synthesis with S^35^ isotope labeling. The read-out was performed by interpreting the tiny electrophoresis bands on autoradiograms and aligning the contiguous segments on a mainframe computer: the monitor was a bland, green-lit CRT screen. We proofread the results by taping standard graph paper into an approximately one yard by one yard sheet—one of these tapestries for each gene segment—and penciling in the day’s results. Needless to say, the entire sequence of the bacteriophage genome took several years to complete, resulting in my first significant publications.

We went on to establish the φ6 genome packaging assay, described in this issue, which was utilized in further studies at PHRI and Helsinki. Lenny encouraged Vesa Olkonnen (visiting from Helsinki) and me to try to recover a recombinant genome segment in a viable φ6 viral particle and we succeeded, setting up the first rescue technology in a segmented dsRNA virus. Lenny, along with the brothers Xueying and Jian Qiao, isolated additional cystoviruses in 1999, greatly expanding the field of study. Lenny cleverly utilized these molecular tools and additional cystovirus types to describe the mechanisms of the viral RNA recombination that is outlined in detail in this Special Issue. His final studies identified host-cell factors that facilitated cystovirus transcription and described with collaborators the crystal structure of capsid protein P1. Lenny was a driving force in the study of virology and is fondly remembered by all those who studied under his guidance and worked alongside him.
